# Fecal dysbiosis associated with colonic hypersensitivity and behavioral alterations in chronically *Blastocystis*-infected rats

**DOI:** 10.1038/s41598-020-66156-w

**Published:** 2020-06-04

**Authors:** Manon Defaye, Céline Nourrisson, Elodie Baudu, Amandine Lashermes, Maëva Meynier, Mathieu Meleine, Ivan Wawrzyniak, Virginie Bonnin, Julie Barbier, Benoit Chassaing, Catherine Godfraind, Agathe Gelot, Nicolas Barnich, Denis Ardid, Mathilde Bonnet, Frédéric Delbac, Frédéric Antonio Carvalho, Philippe Poirier

**Affiliations:** 1 Université Clermont Auvergne, 3iHP, CNRS, Laboratoire Microorganismes: Génome et Environnement, F-63000 Clermont-Ferrand, France; 2 Université Clermont Auvergne, 3iHP, Inserm U1107, NeuroDol, Clermont-Ferrand, France; 3 Université Clermont Auvergne, CHU, 3iHP, CNRS, Laboratoire Microorganismes: Génome et Environnement, F-63000 Clermont-Ferrand, France; 4 Université Clermont Auvergne, 3iHP, Inserm U1071, USC INRA 2018, Microbes, Intestin, Inflammation et Susceptibilité de l’Hôte, Clermont-Ferrand, France; 5 CHU Clermont-Ferrand, Service d’Anatomopathologie, 63003 Clermont-Ferrand, France; 6 Center for Inflammation, Immunity and Infection, Institute for Biomedical Sciences, Georgia State University, Atlanta, GA USA; 7 Neuroscience Institute, Georgia State University, Atlanta, GA USA; 8 INSERM, U1016, team “Mucosal microbiota in chronic inflammatory diseases”, Paris, France; 9 Université de Paris, Paris, France

**Keywords:** Microbiology, Gastroenterology

## Abstract

Background: Infectious gastroenteritis is a risk factor for the development of post-infectious Irritable Bowel Syndrome (PI-IBS). Recent clinical studies reported a higher prevalence of the intestinal parasite *Blastocystis* in IBS patients. Using a rat model, we investigated the possible association between *Blastocystis* infection, colonic hypersensitivity (CHS), behavioral disturbances and gut microbiota changes. Methods: Rats were orally infected with *Blastocystis* subtype 4 (ST4) cysts, isolated from human stool samples. Colonic sensitivity was assessed by colorectal distension and animal behavior with an automatic behavior recognition system (PhenoTyper), the Elevated Plus Maze test and the Forced Swimming tests. Feces were collected at different time points after infection to study microbiota composition by 16 S rRNA amplicon sequencing and for short-chain fatty acid (SFCA) analysis. Results: *Blastocystis*-infected animals had non-inflammatory CHS with increased serine protease activity. Infection was also associated with anxiety- and depressive-like behaviors. Analysis of fecal microbiota composition showed an increase in bacterial richness associated with altered microbiota composition. These changes included an increase in the relative abundance of *Oscillospira* and a decrease in *Clostridium*, which seem to be associated with lower levels of SCFAs in the feces from infected rats. Conclusions: Our findings suggest that experimental infection of rats with *Blastocystis* mimics IBS symptoms with the establishment of CHS related to microbiota and metabolic shifts.

## Introduction

Chronic visceral pain related to colonic hypersensitivity (CHS) is generally described as a poorly localized, diffuse and stabbing sensation that can be associated with many gastrointestinal disorders such as Inflammatory Bowel Disease (IBD) and Irritable Bowel Syndrome (IBS)^1^. IBS generates a significant health care burden, is one of the most common disorders encountered in gastrointestinal practice and greatly affects the quality of life. The Rome IV criteria define IBS as a functional chronic disorder characterized by abdominal pain, changes in bowel habits and no macroscopic organic lesions^2^. Patients are classified into four subgroups: IBS-C for patients with predominant constipation, IBS-D when diarrhea is predominant, IBS-M for patients with alternating constipation and diarrhea and IBS-U when no clear classification can be established^3^. The pathophysiology of IBS is complex and poorly understood, and its etiology is suspected to be multifactorial. IBS patients exhibit several biological perturbations such as disturbances in gut epithelial barrier integrity leading to increased intestinal permeability, immune activation and modifications of intestinal microbiota composition and function^2,4^. Psychological co-morbidities also occur in more than half of cases, such as anxiety, depression and hypochondria^5^.

Infectious gastroenteritis is a key risk factor for the development of IBS, referred to as post-infectious IBS (PI-IBS)^6^. PI-IBS often has characteristics of IBS-D, and can occur in 4% to 31% of patients following infectious acute gastroenteritis^6^. Several studies have supported a role in PI-IBS for pathogen-mediated modifications in the resident intestinal microbiota, epithelial barrier integrity and immune activation^7–9^. The role of certain pathogenic bacteria such as *Shigella* spp., *Escherichia coli*, S*almonella* and *Campylobacter jejuni* or protozoa such as *Giardia intestinalis* is now well established^10^. Interestingly, a recent meta-analysis showed that the risk of IBS was higher after a protozoa-related or parasitic enteritis than with a bacterial infection^11^.

*Blastocystis* spp. are the most frequent enteric protozoa found in the intestinal tract of humans and various animals^12^. These parasites have been classified into 17 subtypes (ST) according to the small subunit ribosomal RNA-encoding gene. Subtypes ST1 to ST9 and ST12 have been recovered in human stool samples with ST3 being the most frequent followed by ST1, ST2 and ST4^13–15^. Interestingly, ST4 was reported to be the most frequent ST in some studies performed in Europe^16–19^. *Blastocystis* spp. prevalence in humans ranges from 0.5% to 100% according to country and hygiene conditions and sanitary practices^13,20^. However, the involvement of *Blastocystis* spp. in human diseases is highly debated. Interest of the scientific and medical communities in *Blastocystis* spp. infection has increased in the last few years since epidemiological studies reported a higher prevalence of the parasite in IBS patients^21–24^. Changes in the microbiota of *Blastocystis*-infected subjects are often reported but results can also be heterogeneous^19,22,25,26^. Some clinical studies reported an increase in microbiota diversity, suggesting a potential benefit for *Blastocystis*-associated microbiota^19,25,26^. In contrast, one other study described a decrease in protective bacteria^22^. A more recent study reported a decrease in short-chain fatty acid (SCFA) levels in fecal samples from *Blastocystis*-infected patients^26^ SCFAs, including acetate, propionate and butyrate, enhance epithelial barrier function and immune tolerance, promoting gut homeostasis^27^.

In the present study we aimed to characterize whether chronic *Blastocystis* infection can lead to IBS-like symptoms. We first characterized non-inflammatory CHS associated with anxiety- and depressive-like behaviors in infected rats. We then demonstrated that *Blastocystis* infection was associated with microbiota and metabolic shifts that can induce intestinal epithelial barrier dysfunction.

## Results

### Blastocystis infection induces non-inflammatory colonic hypersensitivity with an increase in serine protease activity

Four-week-old Wistar rats were infected with 10^5^ ST4 cysts purified from a healthy human carrier (Supplementary Fig. 1). Infection monitoring by xenic culture of the feces showed the presence of parasites from day 1 until euthanasia on day 31 post-infection, as described previously^28^.

**Figure 1:**
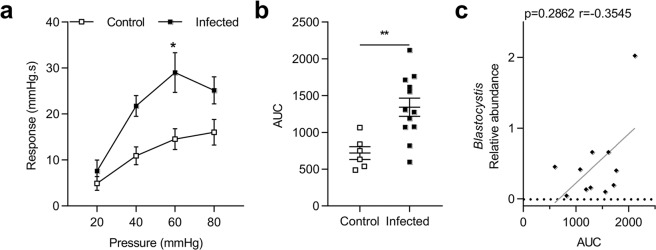
Evaluation of colonic hypersensitivity after infection with *Blastocystis* ST4. (**a**) Representative colonic response to colorectal distension (CRD) in infected (n = 12) and control (n = 6) rats at D31. (**b**) Area under the curve (AUC) of colonic response calculated by the trapeze method between 20 mmHg and 80 mmHg. (**c**) Correlation between relative abundance of *Blastocystis* and AUC of colonic response. Statistical analysis: *a*, Two-way ANOVA test followed by a Sidak *post-hoc* test; *b*, t-test; *c*, Spearman-test; *p < 0.05 **p < 0.01; three independent experiments were performed.

Colorectal distension (CRD) showed that colonic sensitivity was significantly increased at 60 mmHg (p = 0.01) in *Blastocystis* infected rats at D31 (Fig. 1a). AUC from 20 to 80 mmHg were almost 2-times higher in infected rats than in control rats (Fig. 1b).Interestingly, colonic hypersensitivity was not correlated with a relative abundance of *Blastocystis* obtained by 16 S sequencing (Fig. 1c).

**Figure 2:**
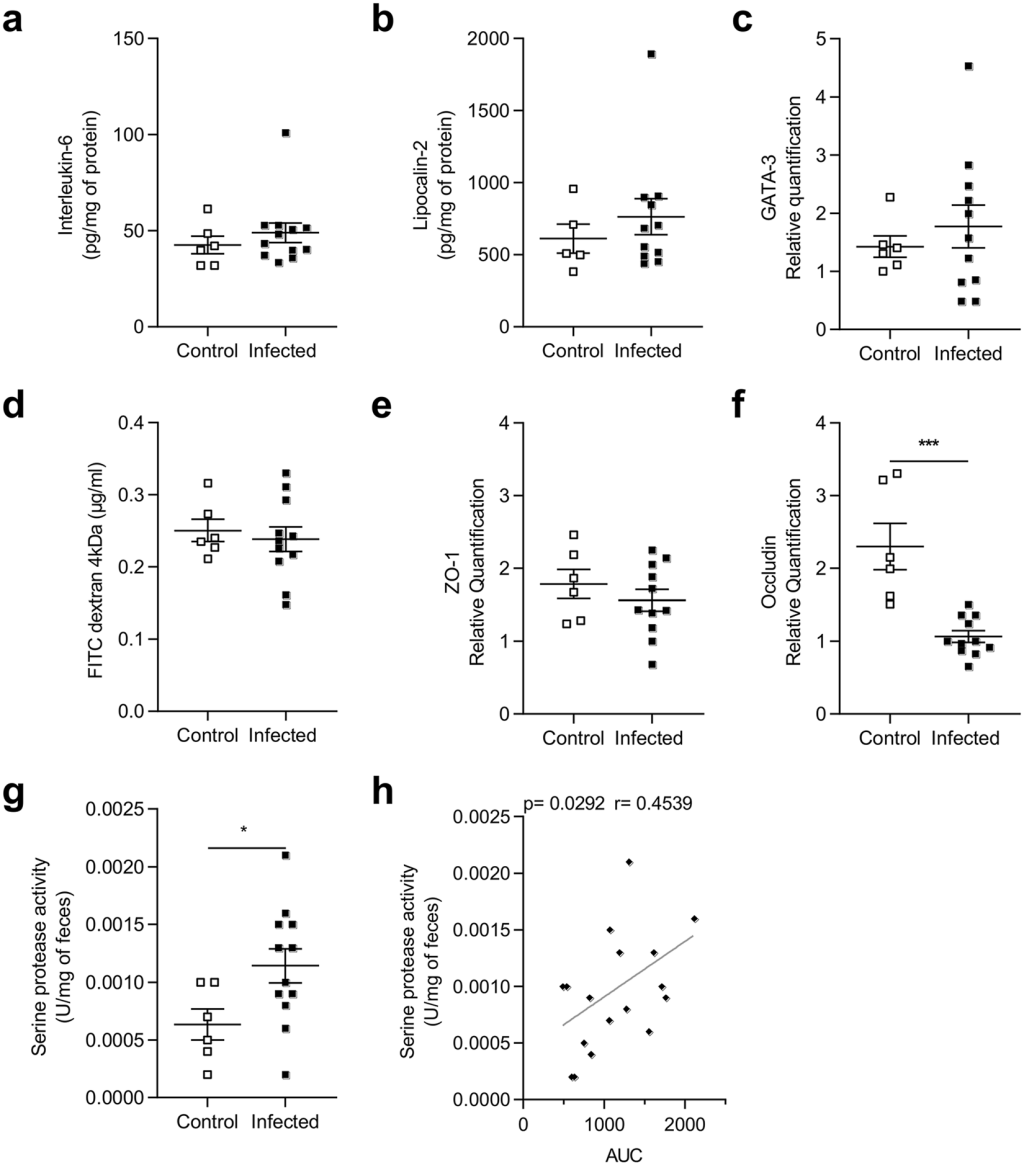
Quantification of inflammatory mediators after infection with *Blastocystis*.(**a,b**) Quantification of Interleukin-6 (IL-6) (**a**) and Lipocalin-2 (**b**) in colonic tissues of control (n = 5) and infected rats (n = 11) at D31. Values were expressed as pg/mg protein. (**c**) Colonic expression of GATA-3 in control (n = 6) and infected rats (n = 11) at D31. (**d**) FITC-Dextran 4 kDa concentration in serum of control (n = 6) and infected (n = 11) rats at D21. Colonic expression of ZO-1 (**e**) and occludin (**f**) in control (n = 6) and infected rats (n = 11) at D31. (**g**) Serine protease activity (U/mg feces) in supernatants from feces of control (n = 6) and infected rats (n = 12) at D31. (**h**) Correlation between serine protease activity and AUC of colonic response. Statistical analysis: Values were expressed as relative expression compared to GAPDH expression. Statistical analysis: *a* and *b*, Mann-Whitney test: *c, d, e, f, g* and *h* t-test; Spearman-test; *p < 0.05 **p < 0.01; ***p < 0.001; three independent experiments were performed.

We then assessed anatomical parameters and inflammatory mediator production at D31 post-infection. Surprisingly, chronic infection had no effect on body weight (Supplementary Fig. 2a), colon weight (Supplementary Fig. 2b) or length (Supplementary Fig. 2c), and was not associated with any histological changes such as cell infiltration, edema or crypt disruption (Supplementary Fig. 2d). Colonic levels of IL-6 and lipocalin-2 proteins were unchanged in infected animals (Fig. 2a,b). In addition, GATA-3 (transcription factor promoting T-helper 2 differentiation) mRNA expression was not higher in infected rats (Fig. 2c).

The potential impact of infection on intestinal barrier integrity was then explored by a functional test using FITC-Dextran 4 kDa on D21 and mRNA quantification of two tight junction components on D31. Intestinal permeability *in vivo* and mRNA levels of ZO-1 were unchanged in the infected animals (Fig. 2d,e) we observed a strong decrease in occludin expression (Fig. 2f). Because intestinal permeability was performed at D21 and a decrease in occludin expression observed at D31, we assessed epithelial barrier function *ex vivo* with Ussing chambers. As previously observed, intestinal permeability was not altered in infected rats (Supplementary Fig. 3).

Serine protease activity, which is suspected to participate in CHS, was measured in fecal samples at D31. A significant increase in serine protease activity was observed in the supernatant of infected rat feces (Fig. 2g). Interestingly, colonic hypersensitivity was significantly correlated with the increase in serine protease activity (p = 0.03) (Fig. 2h).

### *Blastocystis* infection induces behavioral changes

**Figure 3:**
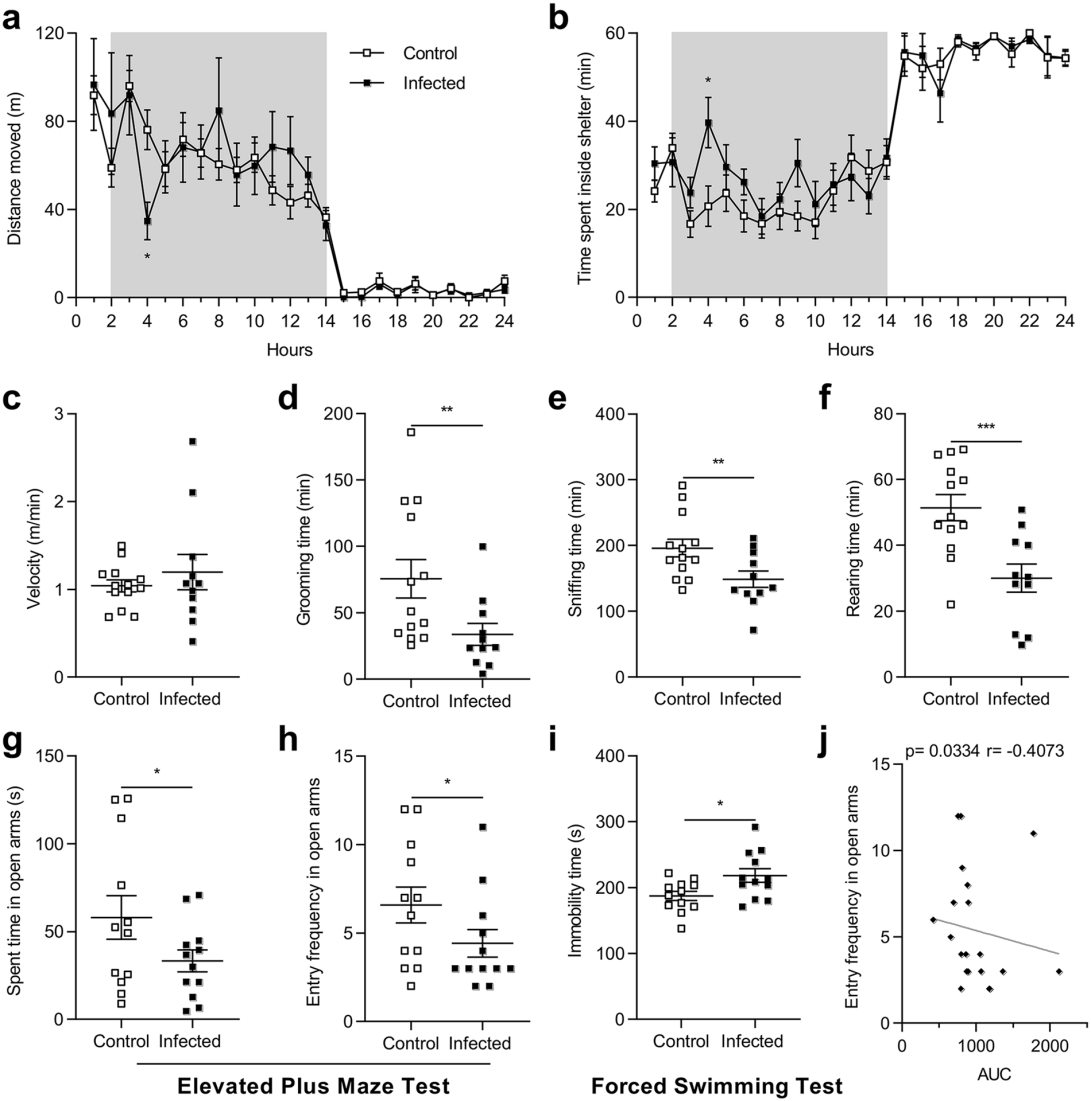
Behavior assessments of rats after infection with *Blastocystis* in a representative experiment. Control and infected rats were placed in PhenoTyper home cages at D27 for 24 h video recording. The total distance moved (**a**) and time spent inside the shelter (**b**) were determined during a 12 h:12 h light-dark cycle. Mean velocity (**c**), grooming (**d**), rearing (**e**) and sniffing (**f**) times were only measured during the 12-hour dark period. Time spent (**g**) and entry frequency (**h**) of control and infected rats into the open arms of the Elevated Plus Maze (EPM) at D29. (**i**) Immobility time during the Forced Swimming Test of control and infected rats at D29. (**j**) Correlation between the areas under the curve (AUC) of colonic sensitivity and the frequency of entries into the open arms of the EPM. Each plot represents one rat. Statistical analyses: *a* and *b*, Two-way ANOVA test followed by a Sidak *post-hoc* test; *c, e, f, g* and *i*, t-test; *d* and *h*, Mann-Whitney; *j*, Spearman-test; *p < 0.05, **p < 0.01, ***p < 0.001; two independent experiments with 12 rats per group were performed.

We analyzed the impact of *Blastocystis* infection on animal behavior at D27 with PhenoTyper. Infected rats did not present any differences in distances traveled (Fig. 3a), time spent in the hidden zone (Fig. 3b), velocity (Fig. 3c), and in drinking and eating behaviors (Supplementary Fig. 4a,b). However, the total duration of grooming (p = 0.004), rearing (p = 0.0006) and sniffing (p = 0.01) was significantly shorter during the dark period (Fig. 3d,e,f) suggestive of anxiety- and depression-like behavior. These behavioral alterations were confirmed at D29 with a series of more classical tests including the Elevated Plus Maze (EPM) test and the Forced Swimming Test (FST). The EPM test showed that infected rats entered the open arms less frequently and spent much less time there (Fig. 3g,h). In addition, immobility time during the FST was longer in infected rats (Fig. 3i), indicative of depressive-like behavior. Interestingly, the AUC value of intracolonic pressure in response to CRD was negatively correlated with the frequency of entry in the open arms (Fig. 3j).

### *Blastocystis* infection is associated with fecal microbiota modifications in rats

**Figure 4:**
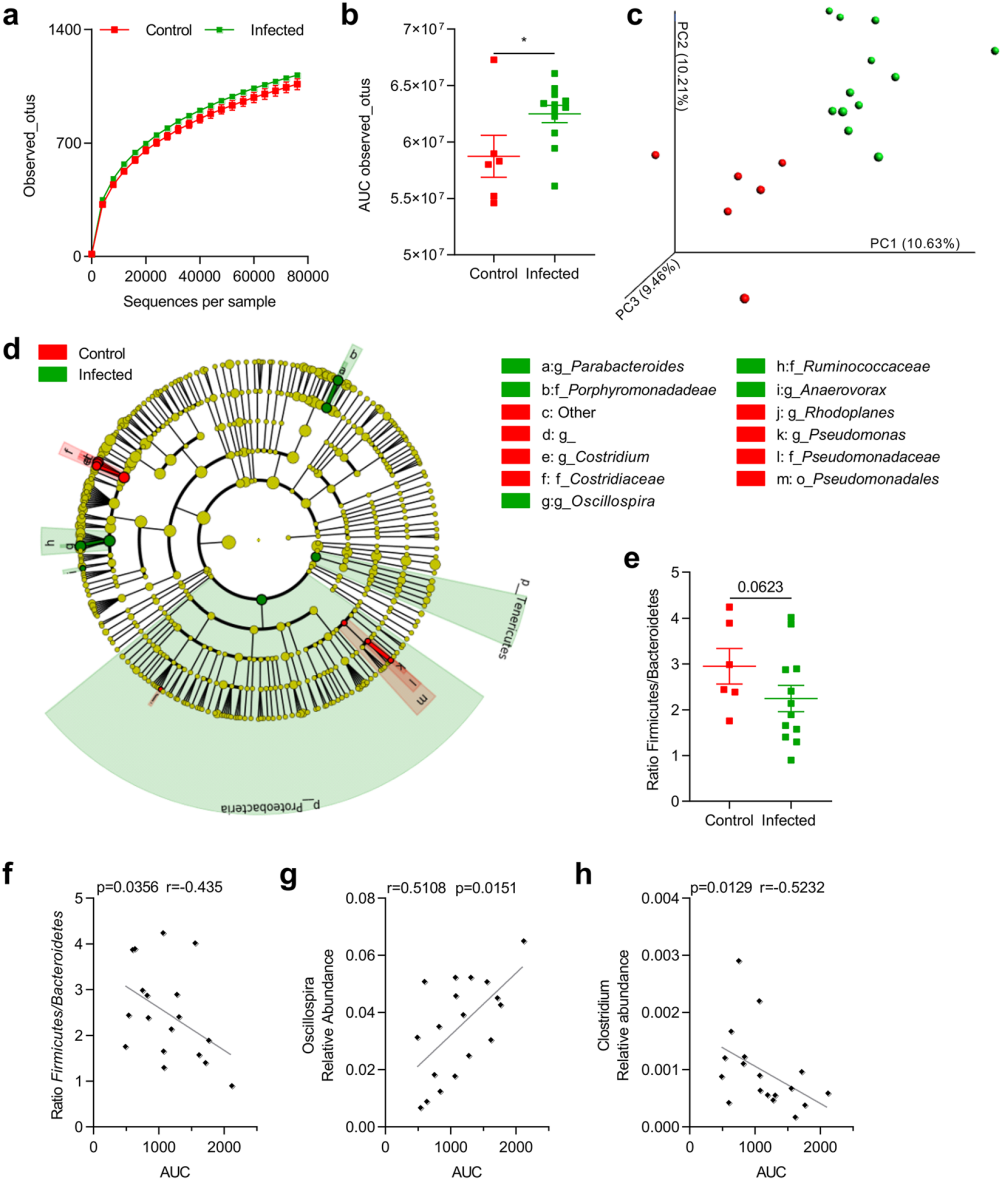
Fecal microbiota variations associated with *Blastocystis* infection in rats in a representative experiment. (**a**) Alpha diversity was determined by observed Operational Taxonomic Unit (OTU) measurement according to sequences per sample in feces of control (n = 6) and infected (n = 12) rats at D31. (**b**) Area under the curve (AUC) of the observed OTUs for control and infected rats. (**c**) Principal coordinates analysis (PCoA) of the unweighted UniFrac distance of control (red plots) and infected (blue plots) rats at D31. Significance (p = 0.001) and the strength of explained variation (R² = 0.0962) were assessed with Adonis. (**d**) LEfSE (LDA Effect Size) was used to investigate bacterial taxa that drive differences between control and infected rats. Red, taxa higher in controls; Green, taxa higher in infected rats. (**e**) Ratio *Firmicutes/Bacteroides* in control (n = 6) and infected (n = 12) rats at D31. (**f**) Correlation between AUC of colonic sensitivity and *Firmicutes/Bacteroides* ratio. (**g**-**h**) Correlation between AUC of colonic sensitivity and relative abundance of *Oscillospira* (**g**) and *Clostridium* (**h**). Statistical analyses: *a* Two-way ANOVA test followed by a Sidak *post-hoc* test; *b*, t-test; *e*, Mann-Whitney; *f*, g and *h*, Spearman-test; *p < 0.05.

To evaluate the influence of *Blastocystis* infection on intestinal microbiota, the fecal microbiota composition of both infected and control rats was characterized by Illumina sequencing (SRA accession PRJNA607663). At D0 (*i.e*. before infection), no difference in fecal microbiota composition was observed between groups (Supplementary Fig. 5). At D31, bacterial richness was significantly greater in chronically-infected rats (p = 0.02) (Fig. 4a,b). In addition, the PCoA of beta-diversity clearly demonstrated significant differences (Adonis, p = 0.001) in the evolution of microbial composition between infected and control rats (Fig. 4c). This was accompanied by an increase in the relative abundance of *Proteobacteria* and *Tenericutes* in infected animals (Fig. 4d). The decrease in the *Firmicutes/Bacteroidetes* ratio (Fig. 4e, p = 0.0623) was correlated with colonic hypersensitivity (p = 0.04) (Fig. 4f). At the genus level, we observed a decrease in the relative abundance of *Clostridium*, *Pseudomonas* and *Rhodoplanes* in infected rats, whereas the relative abundance of *Anaerovorax*, *Oscillospira* and Para*bacteroides* was greater (Fig. 4f). Interestingly, colonic hypersensitivity was significantly correlated with the decrease in the relative abundance of *Oscillospira* (p = 0.0129) (Fig. 4g) and the increase in that of *Clostridium* (p = 0.0151) (Fig. 4h).

**Figure 5:**
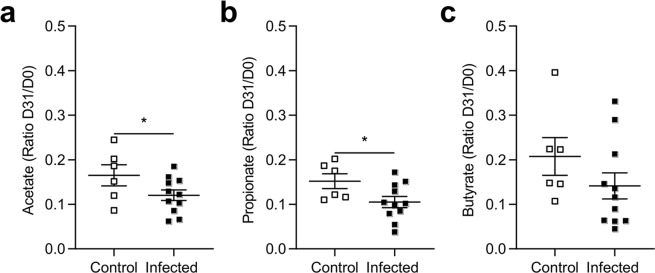
Impact of microbiota modifications on fecal Short-Chain Fatty Acid (SCFA) levels in a representative experiment. D31/D0 ratio of acetate (**a**), propionate (**b**) and butyrate (**c**) in feces from control and infected rats. The SCFAs were quantified by gas liquid chromatography in feces of control (n = 6) and infected rats (n = 12) at D31. Statistical analysis: *a-c*, t-test *p < 0.05.

Shifts in microbiota community composition were associated with metabolite changes in infected rats. We quantified the ratio of SCFAs between the end and beginning of the experiment (D31/D0). We observed a decrease in the D31/D0 ratio for both acetate and propionate (Fig. 5a,b). The decrease in the butyrate ratio was not significant, however, (p = 0.06) (Fig. 5c) in the feces of infected rats.

## Discussion

The role of *Blastocystis* spp. as a human pathogen remains unclear since, although some studies have associated it with acute or chronic digestive disorders^29,30^, it can be found in both symptomatic and asymptomatic patients. In symptomatic patients, blastocystosis is associated with non-specific symptoms, such as chronic diarrhea, abdominal pain and bloating, sometimes mimicking IBS symptoms^31,32^. Interestingly, an increasing number of studies have suggested an association between *Blastocystis* spp. and IBS^21–23^. In numerous studies, *Blastocystis* spp. was more frequently detected in IBS patients than in control groups^21–24^. Experimental and genomic data support this association and suggest interactions between the intestinal microbiota and *Blastocystis* spp., which could be involved in the gut dysbiosis associated with IBS^33^. Our results also suggest that *Blastocystis* spp. chronic infection may lead to IBS-like symptoms. We characterized non-inflammatory CHS associated with anxiety- and depressive-like behaviors in infected rats. We then demonstrated that *Blastocystis* infection was associated with both microbiota and metabolic shifts that can lead to CHS.

The origins of CHS associated with IBS remain elusive. However, CHS occurs in up to 80–90% of IBS patients^34^. Thus, the epidemiological link between *Blastocystis* spp. and IBS needs to be clarified. Using a recent model of chronic infection with *Blastocystis* ST4 in rats, our study establishes for the first time the relationship between *Blastocystis* spp. infection and CHS. As previously described by Leder *et al*., we didn’t correlate the relative abundance with the CHS in infected rats^35^.

During acute gastroenteritis the immune system is highly activated, leading to inflammatory response. After gastroenteritis resolves, inflammation should subside but some studies have described infiltration of immune cells and high cytokine levels in PI-IBS patients, which suggests that the establishment of low grade inflammation following gastroenteritis could be a causal factor of CHS^10^. Some *in vitro* studies have also shown immunomodulatory effects of *Blastocystis* spp. on mammalian cell cultures. The production of pro-inflammatory cytokines, including IL-8, granulocyte-macrophage colony-stimulating factor (GM-CSF), IL-1β, IL-6, and Tumor Necrosis Factor-α (TNF-α) in response to acute *Blastocystis* exposure have been described *in vitro* in epithelial cell lines and murine macrophages^36,37^. In our experimental conditions, we did not observe any clearance of the parasite until 31 days after infection, as observed in PI-IBS, suggesting that the *Blastocystis* infection model is an infectious model of IBS. However, we observed no inflammation or tissue perturbations on D31 (histological analysis, IL-6 and lipocalin-2 levels and GATA-3 mRNA). The disease activity index (DAI) including body weight loss, stool consistency and blood in stool was monitored every week until 31 days post-infection and any modifications were characterized especially in stool consistency. On D21, IL-6 and TNF-α levels in the serum were too low to be quantified in either group.

In absence of inflammation, altered epithelial barrier function is probably one of the most important mechanisms involved in IBS^2^. An increase in intestinal permeability would allow the diffusion of luminal antigens and/or proteases to the submucosal compartments^38^. This intestinal permeability is related to an altered expression of tight junction (TJ) proteins, including a decrease in the expression of occludin and ZO-1^38^. Biopsy analysis from *Blastocystis*-infected patients showed disturbances of barrier function integrity and permeability^39^. These modifications are probably the result of multiple factors, but congruent data have been reported in some *in vitro* experiments showing the ability of *Blastocystis* factors to interfere with ZO-1 and occludin, leading to TJ dysfunctions^40^. In our model, infected animals had lower occludin mRNA expression in the colon and intestinal permeability was not altered. However, study reports that the barrier function of the intestinal epithelium was normal in occludin knockout mice^41^. In our study, the absence of inflammation and barrier impairment could have been due to the use of *Blastocystis* ST4, which is considered to be of murine origin and probably the best suited to rats. Moreover, the isolate used in our study was purified from stools of an asymptomatic human carrier. Interestingly, Hussein *et al*. reported that the degrees of severity of pathological changes and intestinal permeability in rats were correlated with the intensity of symptoms in patients from whom isolates were collected and with STs of *Blastocystis*, suggesting the existence of other, more virulent strains/subtypes^42^.

Hydrolases and proteases have been identified as candidate virulence factors of *Blastocystis* spp. and the panel which are secreted could explain the difference in terms of virulence^33,43,44^. Interestingly, several studies showed an increase in serine protease activity in IBS patients^45–47^. Likewise, higher serine protease activity was observed in a PI-IBS mouse model^48^. Taken together, the data above suggest an important role for serine proteases in IBS. Diffusion to the submucosa of serine proteases, which was increased in our infected rats, could be responsible for activation of the neuronal protease activated receptor (PAR) and nociceptive signal^45^. However, the origin of the serine proteases was unclear. Although *Blastocystis* can produce serine proteases, serine protease activity originates mainly in the host^49^ and the contribution of the gut microbiota to their production is not inconsiderable^43,50^. Further experiments are required to identify this origin and assess how the increase in intra-luminal serine proteases could have contributed to CHS in our model.

Psychological disturbances, especially anxiety and depression, are the most frequent comorbidities associated with visceral pain^51^. In our study, global behavior was analyzed with the PhenoTyper, a video tracking system which allows continuous and long-term monitoring of animals’ home cage behavior. Some behaviors in rats have been reported to be associated with specific psychological or physiological changes. A decrease in grooming is positively associated with depressive-like behavior, and a decrease in sniffing, related to curiosity or exploration, is positively associated with anxiety-like behavior^52,53^. Rearing, which is also considered as a behavior related to exploration, can be an effective indicator of pain^54^. These three behavior profiles were modified in our study and confirmed by EPM and FST reference tests. Our results show that *Blastocystis-*infected rats presented both anxiety- and depressive-like behaviors, probably related to visceral pain. Indeed, we observed a correlation between an increase in time spent in the open arms (EPM test) and CHS severity, which reinforces the role of visceral pain in the behavioral changes seen in our models.

Finally, because gut microbiota is a key player in the pathophysiology of IBS^4^, we characterized the microbiota modifications in *Blastocystis-*infected rats. In the absence of low grade inflammation, colonic hypersensitivity can be explained by alterations in intestinal microbiota composition that result in metabolite changes. Some recent studies described either a healthy *Blastocystis-*associated microbiota characterized by increased bacterial richness and certain beneficial bacteria such as *Akkermansia*, or dysbiosis accompanied by a decrease in SCFA-producing bacteria^19,22,25,26^. A recent study showed that *Blastocystis* ST7 isolate causes changes in the microbiota composition of infected mice, with a decrease in beneficial bacteria^55^, which is consistent with previous results of ours^22^. The association between *Blastocystis* and microbiota composition still needs to be clarified, as does the causal link between microbial community changes and *Blastocystis*. Our results showed that *Blastocystis* ST4 infection in rats is associated with bacterial community shifts, confirming for the first time the effect of this parasite on microbiota composition. The changes were characterized by an increase in bacterial richness and a decrease in the *Firmicutes*/*Bacteroidetes* ratio, which was correlated with CHS. The increase in bacterial richness in human intestinal microbiota associated with *Blastocystis* infection has been reported in different clinical studies, and is usually associated with a healthy microbiota^26^. The *Firmicutes/Bacteroidetes* ratio is a rough indicator of bacterial population shifts, and both a higher and lower ratio of *Firmicutes/Bacteroidetes* have been described in IBS patients^4^. Deeper analysis showed that the relative abundances of bacterial genera were modified in infected animals including an increase in the relative abundance of *Oscillospira* and a decrease in that of *Clostridium*, both being correlated with CHS. Our results are, therefore, in agreement with previous reports describing a decrease in *Clostridium* genus in IBS patients, and the association of that genus with visceral pain^56,57^. The *Clostridiaceae* family, including the *Clostridium* genus, also belongs to SCFA-producing bacteria^58^. SCFAs, including acetate, propionate and butyrate, play an important role in the maintenance of a healthy epithelial barrier, and a decrease in their number has been reported in IBS patients^27,59^. In our study, the feces composition of infected rats was characterized by a decrease in the overall content of SFCAs. Interestingly, a recent study observed a reduction in acetate, propionate and butyrate production in *Blastocystis*-colonized humans, in combination with an over-representation of *Oscillospira*^26^. A recent meta-analysis showed that the fecal level of propionate and butyrate was significantly lower in IBS-C^60^, which is consistent with the absence of epithelial barrier alterations in our study. However, SCFAs might influence neuropsychiatric disorders and psychological functioning as depression^61^. Thus, the decrease of these metabolites may participate to the behavioral disturbance observed in our model.

To conclude, we demonstrate for the first time that establishment of *Blastocystis* within gut microbiota is responsible for bacterial community perturbations associated with metabolic shifts leading to CHS. Interestingly, some of the modifications observed in the infected rats have also been reported in humans^25,26^. The mechanisms of *Blastocystis*-induced CHS are not fully understood but the perturbations observed were sufficient to affect animal behavior. Our results showed that blastocystosis symptoms in rats mimic human IBS, which raises the question of eradication of this protist in *Blastocystis*-positive IBS patients. The *Blastocystis* rat model seems to be a good model to decipher non-inflammatory CHS.

## Materials and Methods

### Animals

Three-week-old specific pathogen-free (Charles River Lab, Saint Germain Nuelles, France) Wistar male rats were housed two or three per cage in animal biosafety level 2 (21–22 °C, 12 h:12 h light-dark cycle) with access to food and water *ad libitum*. All experiments were performed according to the ethical guidelines set out in the Guide for the Care and Use of Laboratory Animals and with approval of the “Comité d’Ethique pour l’Expérimentation Animale Auvergne” (C2E2A), the local ethics committee (Reference number: EU0116–3003), and followed the guidelines of the Committee for Research and Ethical issues of the International Association for the Study of Pain^62^.

### Experimental infections

An asymptomatic human carrier of *Blastocystis* ST4 was enrolled after a statement attesting to informed consent for study participation by the medical laboratory of Parasitology of the Clermont-Ferrand teaching hospital. All experiments were performed in accordance with the relevant guidelines and regulations, and approved by the research ethics committees, “Comité de Protection des Personnes Sud-Est 6”, France, agreement ref: 2014/CE29). Three days prior experiments, rats were pre-screened to ensure that they were healthy and free of *Blastocystis* or any other intestinal parasites. Four-week-old Wistar male rats were then orally inoculated with sterile PBS as control (n = 12) or with 10^5^ *Blastocystis* ST4 cysts purified from human stools (n = 12) as previously reported^28^. The success of infection was confirmed by fecal cultures in Jone’s medium at 37 °C, as previously described^28^. Body weight of animals was monitored and feces were sampled every week until euthanasia and stored at −80 °C. Rats were euthanized at the end of the experiment (D31), and colon tissues were collected and split to be stored at −80 °C for molecular assays or in 4% paraformaldehyde for histological analyses.

### Colorectal distension (CRD) test

This test assesses visceral sensitivity by measuring the response of the colon when submitted to distension. The CRD protocol was adapted for rats from Larauche *et al*.^63^. At D31, after 30 min in a restrainer, rats were anesthetized (3% Isoflurane) to introduce the CRD devices into the colon-rectum before being connected to an amplifier (Millar Instruments, Houston, TX, USA). The CRD protocol consisted of a set of distensions at constant pressures from 20 to 80 mm Hg, performed in duplicate as follows: 20 mm Hg steps of 20 sec duration with 4.5 min inter-stimuli intervals. P-spectrum was extracted from raw data with “smoothsec” at a time constant of 1.5 sec, followed by “absolute valor” and finally another “smoothsec” at a time constant of 1.5 sec to exclude the slower and tonic changes. Colonic response to CRD (mmHg.s) was quantified by measuring the area under the curve (AUC) during 20 sec of distension using the Integral relative to Minimum in Labchart.

### Behavior recognition system PhenoTyper

The PhenoTyper (Noldus Information Technology, Wageningen, The Netherlands) is an automated infrared (IR) video-tracking system developed to measure the behavior of laboratory animals. The device is composed of eight Plexiglas cages (45 cm × 45 cm), each containing an opaque plastic square shelter accessible by two entrances and delimited areas for feeding and drinking. The cages are surrounded by a top unit provided with an infrared (IR) sensitive camera for video recording and IR led units. Briefly, at D27 rats were transferred into PhenoTyper cages (1 per cage) and animal activities were recorded with Mediarecorder (Noldus Information Technology, Wageningen, The Netherlands) for 24 h, including a light-dark cycle (12 h:12 h). Rats had *ad libitum* access to food, water and shelter. Raw data were analyzed with Ethovision XT software (Version 12, Noldus Information Technology, Wageningen, The Netherlands) and various specific behaviors were compared between infected and control rats.

### Elevated-Plus-Maze (EPM) test

At D29, EPM was performed as previously described^64^. Animals were recorded for 5 min by Mediarecorder software. Analysis was performed by Ethovision XT software (version 12).

### Forced Swimming Test (FST)

At D29, each rat was placed in a glass cylinder (30 cm diameter x 50 cm height) containing water at 22 ± 1 °C. The immobility time of rats was recorded for 6 min. Each rat was judged to be immobile when it stopped swimming and floated, making only those movements necessary to keep its head above water^65^.

### *In vivo* intestinal permeability assay

At D21 intestinal permeability was determined by FITC-dextran in fasted animals as previously described^66^. Briefly, rats were administered 60 mg/100 g body weight of FITC-dextran (4 kDa, Sigma-Aldrich, FD-4) by gavage and samples were obtained from the retro-orbital venous plexus 5 h after administration. Serum FITC levels were determined by fluorometry at 488 nm using a microplate reader (Tecan, Lyon, France). Concentrations were calculated from a standard curve.

### Assessment of epithelial barrier function

Two segments of distal colon of each animal were mounted in modified Ussing chambers (Biomecatronics, Ruitz, France) after removal of the seromuscular layer, leaving an exposed area of 0.0314 cm^2^. The luminal and basolateral compartments were filled with Krebs‐Ringer bicarbonate buffer (NaCl 120 mM, KCl: 5.9 mM, MgCl2: 1.2 mM, NaH2PO4: 1.2 mM, CaCl2: 2.5 mM, NaHCO3: 14 mM) supplemented with 10 mmol/L glucose. Solutions were kept at 37 °C and gassed with carbogen. Transmucosal potential difference was continuously monitored using calomel electrodes filled with saturated KCl and connected to the chambers with 3 M KCl-agar salt-bridges. The transepithelial electrical resistance (TEER) was calculated according to Ohm’s law from the voltage deflections induced by bipolar current pulses of 10 µA with a duration of about 2 sec. The TEER values were registered for each tissue just after mounting, at 40 minutes after mounting and then at 30‐minute intervals up to 160 minutes. The average TEER between 100 and 160 minutes was calculated over the two pieces of tissue per animal. These time points were selected because a stable TEER plateau was reached in most of the tissues at these times.

### Tissue preparation for histological analysis

Flushed colons were opened longitudinally, cut into 4 cm and rolled. The samples were fixed in 4% paraformaldehyde (24 h, 4 °C), incubated in 30% sucrose (48 h, 4 °C) and embedded in OCT medium (ThermoFisher Scientific, Waltham, MA, USA; Cat. No. 23-730-571). Ten micrometer cross-sections were mounted on SuperFrost Plus slides (ThermoFisher Scientific, Waltham, MA, USA; Cat. No. 10149870), stained with hematoxylin phloxin safran and examined blindly by an anatomopathologist according to the following criteria: cellular infiltration and mucosal alteration (vasculitis, muscular thickening, and crypt abscesses) were graded from 0 to 3 (absent, mild, moderate and severe). Submucosal edema was scored from 0 to 2 (absent, moderate, and severe).

### Enzyme-linked immunosorbent assay (ELISA)

Total proteins of rat proximal colon were extracted with lysis buffer (50 mM HEPES, 150 mM NaCl, 10 mM EDTA, 10 mM tetrasodium pyrophosphate, 2 mM vanadate, 100 mM NaF, 0.5 mM PMSF, 100 UI/mL iniprol/aprotinin, 20 µM leupeptin, 1% triton), and protein concentrations were determined with a BC Assay Protein quantification kit (Interchim, Montluçon, France; Cat. No. UP40840A). Colonic interleukin-6 (IL-6) and lipocalin-2 were quantified with the enzyme linked immunosorbent assay kit (R&D Systems, Minneapolis, MN, USA; Cat. No. Rat IL-6 DuoSet, DY506 and Rat Lipocalin-2/NGAL DuoSet DY3508) according to the manufacturer’s instructions. Concentrations were calculated from standard curve and normalized to the total protein concentration.

### Reverse transcription and quantitative polymerase chain reaction (RT-qPCR)

Total RNAs of proximal colon were extracted with Trizol (ThermoFisher Scientific, Waltham, MS, USA; Cat. No. 15596026). DNAse-treated RNAs were reverse transcribed with high capacity cDNA RT kit (ThermoFisher Scientific, Waltham, MS, USA; Cat. No. 4368814) for reverse transcription-quantitative PCR (RT-qPCR). Specific cDNA were amplified for occludin (occF 5′-AGTACATGGCTGCTGCTGATG-3′; occR 5′-CCCACCATCCTCTTGATGTGT-3′), *zonula occludens-*1 (ZO-1) (ZO-1F 5′-AGCGAAGCCACCTGAAGATA-3′; ZO-1R 5′-GATGGCCAGCAGGAATATGT-3′), GATA-3 (GATA-3F 5′-AAGAGTGCCTCAAGTATCAG-3′; GATA-3R 5′- GCGGATAGGTGGTAATGG-3′) and GAPDH (GAPDHF 5′-AGACAGCCGCATCTTCTTGT-3′; GAPDHR 5′-TGATGGCAACAATGTCCACT-3′). qPCR assays were performed with SsoAdvanced Universal SYBR Green Supermix (Biorad, Hercules, CA, USA; Cat. No. 1725271) and carried out on CFX96 Touch Real-Time PCR Detection System (Biorad, Hercules, CA, USA). Relative quantifications of occludin, ZO-1 and GATA-3 genes were expressed as fold-change, using the 2^−∆∆Ct^ method with GAPDH as reference gene.

### Fecal microbiota analysis

DNA was extracted from rat feces collected at D0 and D31 with the NucleoSpin Soil kit protocol (Macherey-Nagel SARL, Hoerdt, France; Cat. No. 740780.250). Illumina high throughput sequencing was performed by MRDNA lab (Shallowater, TX, USA) on a MiSeq following the manufacturer’s guidelines. Briefly, the V4 region of the bacterial 16 S rRNA gene was amplified by 515 F (5′-GTGCCAGCMGCCGCGGTAA-3′) and 806 R (5′-GGACTACHVGGGTWTCTAAT-3′) primers and HotStarTaq Plus Master Mix Kit (Qiagen, Germantown, MD, USA; Cat. No. 203646) under the following conditions: denaturation 94 °C/3 min, followed by 28 cycles of 94 °C/30 sec, 53 °C/40 sec, and 72 °C/1 min, with a final elongation step 72 °C/5 min. Pooled PCR products were purified with calibrated Ampure XP beads. Illumina sequencing was performed and DNA libraries built by according to the Illumina TruSeq DNA library preparation protocol.

We performed microbiota analysis on Quantitative Insights Into Microbial Ecology (QIIME, version 1.8.0) software package^67^. In summary, sequences were demultiplexed to remove barcodes and primer sequences. Chimeric sequences were removed with USEARCH61^68^. Sequences were clustered with USEARCH61 at a 97% homology threshold^69^. Taxonomic analysis was performed with the Greengenes reference database (version 13-8).

Alpha diversity measures the richness of single microbial taxa within a sample. Observed operational taxonomic unit (OTU) measurements were determined with QIIME using an OTU table rarefied at various depths. AUC were calculated for each rarefaction curve. Beta diversity measures the variation in microbiota composition between individual samples. Unweighted UniFrac distances between samples were computed to measure beta diversity with the rarefied OTUs count table. Principal coordinates analysis (PCoA) was used to further assess and visualize beta diversity. Groups were compared for distinct clustering with Adonis. LEfSe (LDA Effect Size) was used to investigate bacterial members that drive differences between groups^70^.

### Short chain fatty acid (SCFA) analysis

Acetate, propionate and butyrate were quantified from rat feces collected at D0 and D31. Briefly, about 200 mg of fecal samples were diluted in 200 µl of distilled water. After centrifugation, proteins were precipitated overnight with phosphotungstic acid (Sigma-Aldrich, Saint-Louis, MO, USA, Cat. No. P4006) and centrifuged to obtain a clear supernatant.

One microliter of the clear supernatant was used to analyze SCFA composition by gas liquid chromatography (Agilent technologies 6850 Network GC system, Santa Clara, SA, USA) with a splitless injector, a flame-ionization detector and a capillary column (30 m; 0.25 mm; 0.25 µm) impregnated with nitroterephthalic acid modified polyethylene glycol (Agilent technologies, Santa Clara, SA, USA; Cat. No. AT-122-3232E). Carrier gas (Helium) flow rate was 0.7 ml/min and inlet, column and detector temperatures were 175, 100 and 240 °C, respectively. Volatile free acid mix was used as the internal standard (Supelco. Saint-Quentin-Fallavier, France; Cat. No. CRM46975).

Data were collected and peaks integrated with OpenLAB software (Agilent technologies, Santa Clara, SA, USA). Values were expressed in M/g of feces and the ratio was calculated between D31 and D0.

### Serine protease activity assay

About 200 mg of feces were diluted in 1 ml of distilled water, homogenized and centrifuged at 1000 g for 5 min. One hundred microliters of fecal supernatants were added to 100 µl of buffer (50 mM Tris-HCl pH 8, 1 mM CaCl_2_) containing 100 µM specific serine protease substrate (suc-phe-ala-ala-phe-pNA) (Bachem, Switzerland; Cat. No. 4013859) and incubated at 37 °C for 6 h. Substrate cleavage was measured at 400 nm with NUNC 96-well plates (ThermoFisher Scientific, Waltham, MS, USA) and normalized to the total feces weight.

### Statistical analysis

Statistical analysis was performed with Prism 7 software (GraphPad, La Jolla, CA, USA). Data were expressed as mean ± standard error of mean (SEM). Colonic sensitivity to gradual CRD, body weight, distance moved, time spent in the hidden zone and microbiota analysis (taxonomy) were analyzed by two-way ANOVA followed by Sidak *post hoc* test for multiple comparisons. Rate comparisons were performed by Fisher’s exact test. Most comparisons were performed by the Student test. For non-Gaussian data, comparisons were performed by the non-parametric Mann Whitney U test (Unpaired data). A p-value ≤ 0.05 was considered statistically significant.

## Supplementary information

Supplementary information.

## References

[1] Johnson AC, Greenwood-Van Meerveld B (2016). The Pharmacology of Visceral Pain. in. Advances in pharmacology (San Diego, Calif.).

[2] Enck P (2016). Irritable bowel syndrome. Nat. Rev. Dis. Prim..

[3] Drossman DA (2016). Functional gastrointestinal disorders: what’s new for Rome IV?. Lancet Gastroenterol. Hepatol..

[4] Rodiño-Janeiro BK, Vicario M, Alonso-Cotoner C, Pascua-García R, Santos J (2018). A Review of Microbiota and Irritable Bowel Syndrome: Future in Therapies. Adv. Ther..

[5] Fond G (2014). Anxiety and depression comorbidities in irritable bowel syndrome (IBS): a systematic review and meta-analysis. Eur. Arch. Psychiatry Clin. Neurosci..

[6] Lee YY, Annamalai C, Rao SSC (2017). Post-Infectious Irritable Bowel Syndrome. Curr. Gastroenterol. Rep..

[7] Ohman L, Simrén M (2010). Pathogenesis of IBS: role of inflammation, immunity and neuroimmune interactions. Nat. Rev. Gastroenterol. Hepatol..

[8] Bercík P (2004). Visceral hyperalgesia and intestinal dysmotility in a mouse model of postinfective gut dysfunction. Gastroenterology.

[9] Beatty JK, Bhargava A, Buret AG (2014). Post-infectious irritable bowel syndrome: mechanistic insights into chronic disturbances following enteric infection. World J. Gastroenterol..

[10] Downs IA, Aroniadis OC, Kelly L, Brandt LJ (2017). Postinfection Irritable Bowel Syndrome. J. Clin. Gastroenterol..

[11] Klem F (2017). Prevalence, Risk Factors, and Outcomes of Irritable Bowel Syndrome After Infectious Enteritis: A Systematic Review and Meta-analysis. Gastroenterology.

[12] Tan KSW (2008). New Insights on Classification, Identification, and Clinical Relevance of Blastocystis spp. Clin. Microbiol. Rev..

[13] Alfellani MA (2013). Variable geographic distribution of Blastocystis subtypes and its potential implications. Acta Trop..

[14] Rene BA, Stensvold CR, Badsberg JH, Nielsen HV (2009). Subtype analysis of Blastocystis isolates from Blastocystis cyst excreting patients. *Am*. J. Trop. Med. Hyg..

[15] Ramírez JD (2016). Geographic distribution of human Blastocystis subtypes in South America. Infect. Genet. Evol..

[16] Poirier P (2011). Development and Evaluation of a Real-Time PCR Assay for Detection and Quantification of Blastocystis Parasites in Human Stool Samples: Prospective Study of Patients with Hematological Malignancies. J. Clin. Microbiol..

[17] Domínguez-Márquez MV, Guna R, Muñoz C, Gómez-Muñoz MT, Borrás R (2009). High prevalence of subtype 4 among isolates of Blastocystis hominis from symptomatic patients of a health district of Valencia (Spain). Parasitol. Res..

[18] Olsen KEP, Christiansen DB, Nielsen HV, Stensvold CR (2011). Blastocystis sp. Subtype 4 is Common in Danish Blastocystis-Positive Patients Presenting with Acute Diarrhea. Am. J. Trop. Med. Hyg..

[19] Tito Raul Y, Chaffron Samuel, Caenepeel Clara, Lima-Mendez Gipsi, Wang Jun, Vieira-Silva Sara, Falony Gwen, Hildebrand Falk, Darzi Youssef, Rymenans Leen, Verspecht Chloë, Bork Peer, Vermeire Severine, Joossens Marie, Raes Jeroen (2018). Population-level analysis of Blastocystis subtype prevalence and variation in the human gut microbiota. Gut.

[20] El Safadi D (2014). Children of Senegal River Basin show the highest prevalence of Blastocystissp. ever observed worldwide. BMC Infect. Dis..

[21] Ragavan, N. D., Kumar, S., Chye, T. T., Mahadeva, S. & Shiaw-Hooi, H. Blastocystis sp. in Irritable Bowel Syndrome (IBS) - Detection in Stool Aspirates during Colonoscopy. *Plos one* **10**, e0121173 (2015). 10.1371/journal.pone.0121173 PMC4572711 26375823

[22] Nourrisson Céline, Scanzi Julien, Pereira Bruno, NkoudMongo Christina, Wawrzyniak Ivan, Cian Amandine, Viscogliosi Eric, Livrelli Valérie, Delbac Frédéric, Dapoigny Michel, Poirier Philippe (2014). Blastocystis Is Associated with Decrease of Fecal Microbiota Protective Bacteria: Comparative Analysis between Patients with Irritable Bowel Syndrome and Control Subjects. PLoS ONE.

[23] Rostami A (2017). Erratum to: the role of Blastocystis sp. and Dientamoeba fragilis in irritable bowel syndrome: a systematic review and meta-analysis. Parasitol. Res..

[24] Kesuma Y, Firmansyah A, Bardosono S, Sari IP, Kurniawan A (2019). Blastocystis ST-1 is associated with Irritable Bowel Syndrome-diarrhoea (IBS-D) in Indonesian adolescences. Parasite Epidemiol. Control.

[25] Audebert C (2016). Colonization with the enteric protozoa Blastocystis is associated with increased diversity of human gut bacterial microbiota. Sci. Rep..

[26] Nieves-Ramírez, M. E. *et al*. Asymptomatic Intestinal Colonization with Protist Blastocystis Is Strongly Associated with Distinct Microbiome Ecological Patterns. *mSystems* **3** (2018). 10.1128/mSystems.00007-18 PMC6020473 29963639

[27] Rooks MG, Garrett WS (2016). Gut microbiota, metabolites and host immunity. Nat. Rev. Immunol..

[28] Defaye Manon, Nourrisson Céline, Baudu Elodie, Warwzyniak Ivan, Bonnin Virginie, Bonnet Mathilde, Barnich Nicolas, Ardid Denis, Delbac Frédéric, Carvalho Frédéric Antonio, Poirier Philippe (2018). Efficient and reproducible experimental infections of rats with Blastocystis spp. PLOS ONE.

[29] Cekin AH (2012). Blastocystosis in patients with gastrointestinal symptoms: a case–control study. BMC Gastroenterol..

[30] Stark D, van Hal S, Marriott D, Ellis J, Harkness J (2007). Irritable bowel syndrome: a review on the role of intestinal protozoa and the importance of their detection and diagnosis. Int. J. Parasitol..

[31] Toro Monjaraz EM (2018). Blastocystis Hominis and Chronic Abdominal Pain in Children: Is there an Association between Them?. J. Trop. Pediatr..

[32] Salvador F (2016). Epidemiological and clinical profile of adult patients with Blastocystis sp. infection in Barcelona, Spain. Parasit. Vectors.

[33] Poirier P, Wawrzyniak I, Vivarès CP, Delbac F, El Alaoui H (2012). New insights into Blastocystis spp.: A potential link with irritable bowel syndrome. PLoS Pathog..

[34] Farzaei MH, Bahramsoltani R, Abdollahi M, Rahimi R (2016). The Role of Visceral Hypersensitivity in Irritable Bowel Syndrome: Pharmacological Targets and Novel Treatments. J. Neurogastroenterol. Motil..

[35] Leder K, Hellard ME, Sinclair MI, Fairley CK, Wolfe R (2005). No correlation between clinical symptoms and Blastocystis hominis in immunocompetent individuals. J. Gastroenterol. Hepatol..

[36] Puthia MK, Lu J, Tan KSW (2008). Blastocystis ratti contains cysteine proteases that mediate interleukin-8 response from human intestinal epithelial cells in an NF-kappaB-dependent manner. Eukaryot. Cell.

[37] Lim MX (2014). Differential regulation of proinflammatory cytokine expression by mitogen-activated protein kinases in macrophages in response to intestinal parasite infection. Infect. Immun..

[38] Camilleri M (2012). Intestinal barrier function in health and gastrointestinal disease. Neurogastroenterol. Motil..

[39] Dagci, H. *et al*. Epidemiological and diagnostic features of blastocystis infection in symptomatic patients in izmir province, Turkey. *Iran. J. Parasitol*. **9**, 519–29 PMC4345091 25759733

[40] Nourrisson C (2016). On Blastocystis secreted cysteine proteases: a legumain-activated cathepsin B increases paracellular permeability of intestinal Caco-2 cell monolayers. Parasitology.

[41] Saitou M (2000). Complex phenotype of mice lacking occludin, a component of tight junction strands. Mol. Biol. Cell.

[42] Hussein EM, Hussein AM, Eida MM, Atwa MM (2008). Pathophysiological variability of different genotypes of human Blastocystis hominis Egyptian isolates in experimentally infected rats. Parasitol. Res..

[43] Denoeud F (2011). Genome sequence of the stramenopile Blastocystis, a human anaerobic parasite. Genome Biol..

[44] Gentekaki Eleni, Curtis Bruce A., Stairs Courtney W., Klimeš Vladimír, Eliáš Marek, Salas-Leiva Dayana E., Herman Emily K., Eme Laura, Arias Maria C., Henrissat Bernard, Hilliou Frédérique, Klute Mary J., Suga Hiroshi, Malik Shehre-Banoo, Pightling Arthur W., Kolisko Martin, Rachubinski Richard A., Schlacht Alexander, Soanes Darren M., Tsaousis Anastasios D., Archibald John M., Ball Steven G., Dacks Joel B., Clark C. Graham, van der Giezen Mark, Roger Andrew J. (2017). Extreme genome diversity in the hyper-prevalent parasitic eukaryote Blastocystis. PLOS Biology.

[45] Cenac N (2007). Role for protease activity in visceral pain in irritable bowel syndrome. J. Clin. Invest..

[46] Róka R (2007). A pilot study of fecal serine-protease activity: a pathophysiologic factor in diarrhea-predominant irritable bowel syndrome. Clin. Gastroenterol. Hepatol..

[47] Gecse K (2008). Increased faecal serine protease activity in diarrhoeic IBS patients: a colonic lumenal factor impairing colonic permeability and sensitivity. Gut.

[48] Ibeakanma C (2011). Brain–Gut Interactions Increase Peripheral Nociceptive Signaling in Mice With Postinfectious Irritable Bowel Syndrome. Gastroenterology.

[49] Tooth D (2014). Characterisation of faecal protease activity in irritable bowel syndrome with diarrhoea: origin and effect of gut transit. Gut.

[50] Steck N, Mueller K, Schemann M, Haller D (2012). Bacterial proteases in IBD and IBS. Gut.

[51] Shah E, Rezaie A, Riddle M, Pimentel M (2014). Psychological disorders in gastrointestinal disease: epiphenomenon, cause or consequence?. Ann. Gastroenterol..

[52] Rosa PB (2014). Folic acid prevents depressive-like behavior induced by chronic corticosterone treatment in mice. Pharmacol. Biochem. Behav..

[53] Crumeyrolle-Arias M (2014). Absence of the gut microbiota enhances anxiety-like behavior and neuroendocrine response to acute stress in rats. Psychoneuroendocrinology.

[54] Cho H (2013). Voluntary movements as a possible non-reflexive pain assay. Mol. Pain.

[55] Yason JA, Liang YR, Png CW, Zhang Y, Tan KSW (2019). Interactions between a pathogenic Blastocystis subtype and gut microbiota: *in vitro* and *in vivo* studies. Microbiome.

[56] X.-Y. Z (2016). Visceral hypersensitive rats share common dysbiosis features with irritable bowel syndrome patients. World J. Gastroenterol..

[57] Zhao K, Yu L, Wang X, He Y, Lu B (2018). Clostridium butyricum regulates visceral hypersensitivity of irritable bowel syndrome by inhibiting colonic mucous low grade inflammation through its action on NLRP6. Acta Biochim. Biophys. Sin. (Shanghai)..

[58] Hugenholtz Floor, Davids Mark, Schwarz Jessica, Müller Michael, Tomé Daniel, Schaap Peter, Hooiveld Guido J. E. J., Smidt Hauke, Kleerebezem Michiel (2018). Metatranscriptome analysis of the microbial fermentation of dietary milk proteins in the murine gut. PLOS ONE.

[59] Pozuelo M (2015). Reduction of butyrate- and methane-producing microorganisms in patients with Irritable Bowel Syndrome. Sci. Rep..

[60] Sun Q, Jia Q, Song L, Duan L (2019). Alterations in fecal short-chain fatty acids in patients with irritable bowel syndrome: A systematic review and meta-analysis. Medicine (Baltimore)..

[61] Dalile B, Van Oudenhove L, Vervliet B, Verbeke K (2019). The role of short-chain fatty acids in microbiota–gut–brain communication. Nature Reviews Gastroenterology and Hepatology.

[62] Zimmermann M (1983). Ethical guidelines for investigations of experimental pain in conscious animals. Pain.

[63] Larauche M, Gourcerol G, Million M, Adelson DW, Taché Y (2010). Repeated psychological stress-induced alterations of visceral sensitivity and colonic motor functions in mice: influence of surgery and postoperative single housing on visceromotor responses. Stress.

[64] Aissouni Y (2017). Acid-Sensing Ion Channel 1a in the amygdala is involved in pain and anxiety-related behaviours associated with arthritis. Sci. Rep..

[65] Slattery DA, Cryan JF (2012). Using the rat forced swim test to assess antidepressant-like activity in rodents. Nat. Protoc..

[66] Tulstrup Monica Vera-Lise, Christensen Ellen Gerd, Carvalho Vera, Linninge Caroline, Ahrné Siv, Højberg Ole, Licht Tine Rask, Bahl Martin Iain (2015). Antibiotic Treatment Affects Intestinal Permeability and Gut Microbial Composition in Wistar Rats Dependent on Antibiotic Class. PLOS ONE.

[67] Caporaso JG (2010). QIIME allows analysis of high-throughput community sequencing data. Nat. Methods.

[68] Edgar RC, Flyvbjerg H (2015). Error filtering, pair assembly and error correction for next-generation sequencing reads. Bioinformatics.

[69] Edgar RC (2010). Search and clustering orders of magnitude faster than BLAST. Bioinformatics.

[70] Segata Nicola, Izard Jacques, Waldron Levi, Gevers Dirk, Miropolsky Larisa, Garrett Wendy S, Huttenhower Curtis (2011). Metagenomic biomarker discovery and explanation. Genome Biology.

