# Supplementary material for: Fecal dysbiosis associated with colonic hypersensitivity and behavioral alterations in chronically Blastocystis-infected rats
Source: Sci Rep. 2020 Jun 4;10:9146. doi: 10.1038/s41598-020-66156-w (PMC7272397; doi:10.1038/s41598-020-66156-w)
Supplement: Supplementary file 1 — Supplementary information. [file 41598_2020_66156_MOESM1_ESM.pdf]

Fecal dysbiosis associated with colonic hypersensitivity and behavioral alterations in chronically *Blastocystis*-infected rats

Manon Defaye, Céline Nourrisson, Elodie Baudu, Amandine Lashermes, Maëva Meynier, Mathieu Meleine, Ivan Wawrzyniak, Virginie Bonnin, Julie Barbier, Benoit Chassaing, Catherine Godfraind, Agathe Gelot, Nicolas Barnich, Denis Ardid, Mathilde Bonnet, Frédéric Delbac, Frédéric Antonio Carvalho and Philippe Poirier

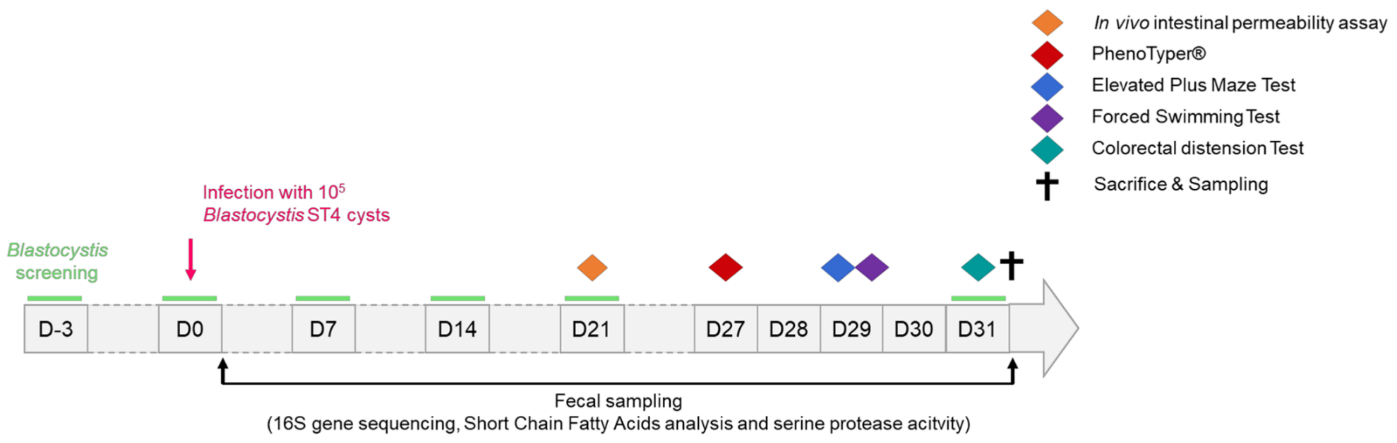

Supplementary Figure 1

Flow chart of experimental protocol. After *Blastocystis* spp. screening at 3 days before infection (D-3), 4-week-old male Wistar rats received either 10^5 *Blastocystis* ST4 cysts in sterile PBS (n=12) or only sterile PBS (control, n=12) by orogastric gavage at D0. Freshly dropped fecal pellets were collected at D0, D7, D14, D21 and D31 (black arrows) to monitor infection (*Blastocystis* screening - green line), fecal microbiota composition (through 16S rRNA gene sequencing), short-chain fatty acid (SCFA) analysis and serine protease activity. Intestinal permeability was measured at D21 with FITC-dextran 4kDa. *Behavioral profile of rats* was assessed at D27 with PhenoTyper and at D29 with Elevated Plus Maze (EPM) test and Forced Swimming Test (FST). At D31 a colorectal distension (CRD) test was performed after which all animals were euthanized and their colon collected to analyze inflammatory mediators and tight junction expression.

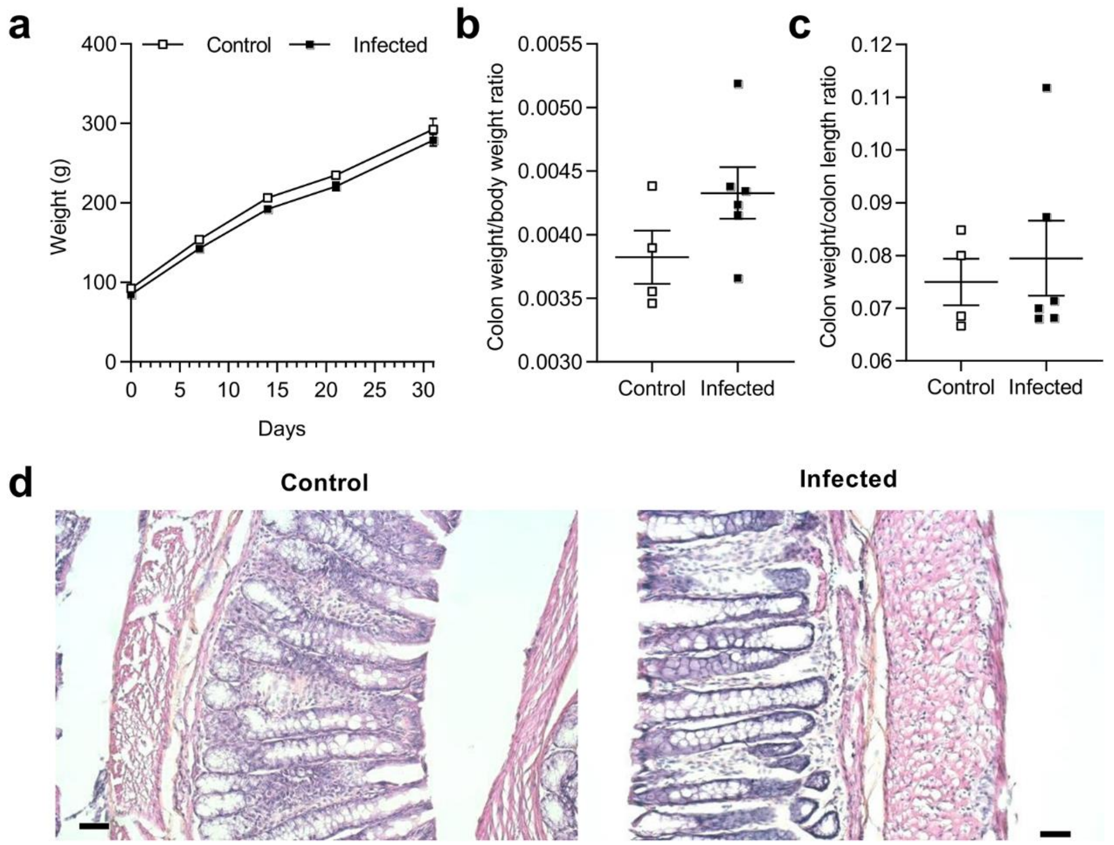

Supplementary Figure 2

Evaluation of anatomical parameters. (**a**) Body weight monitoring of control (n=6) and infected (n=12) rats from D0 to D31. (**b**) Colon weight/body weight ratio of control (n=4) and infected (n=6) rats at D31. (**c**) Colon weight/length ratio of control (n=4) and infected (n=6) rats at D31. (**d**) Representative colonic tissue sections of control (n=6) and infected (n=11) rats at D31. Scale bar: 10 µm. Statistical analysis: *a*, Two-way ANOVA test followed by a Sidak *post-hoc* test; *b*, t-test; *c*, Mann-Whitney test; * $p<0.05$, ** $p<0.01$, *** $p<0.001$; three independent experiments were performed.

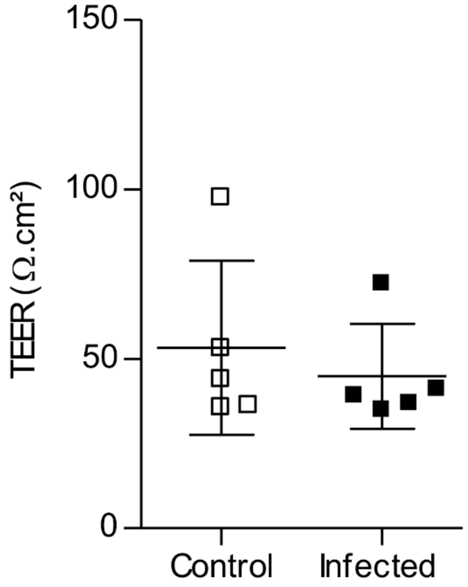

Supplementary Figure 3

Barrier function was evaluated with Ussing-chambers in the distal colon of control (n=5) and infected rats (n=5). Transmural resistance (TERR) was measured for 160 min after mucosa mounting in the chamber. The average TEER between 100 and 160 minutes was calculated over the two pieces of tissue per animal.

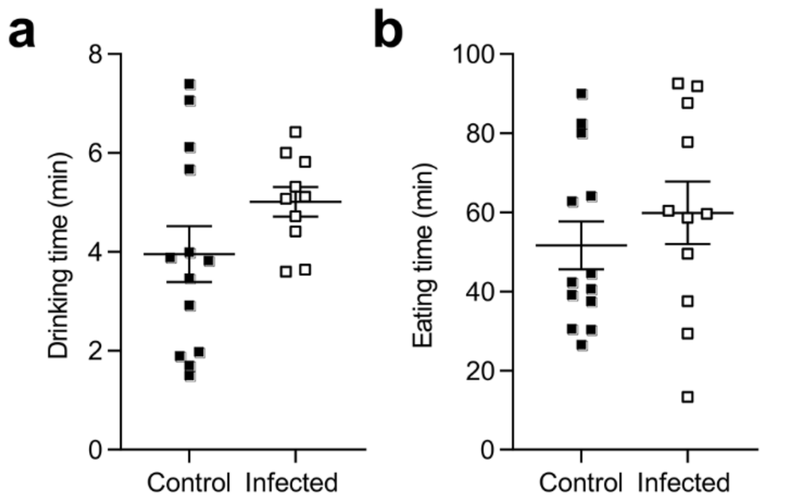

Supplementary Figure 4
Behavioral analysis by PhenoTyper. Drinking (**a**) and eating (**b**) times during the 12-hour dark period for control (n=13) and infected (n=11) rats at D27. Statistical analysis was performed with the t-test. Two independent experiments were performed.

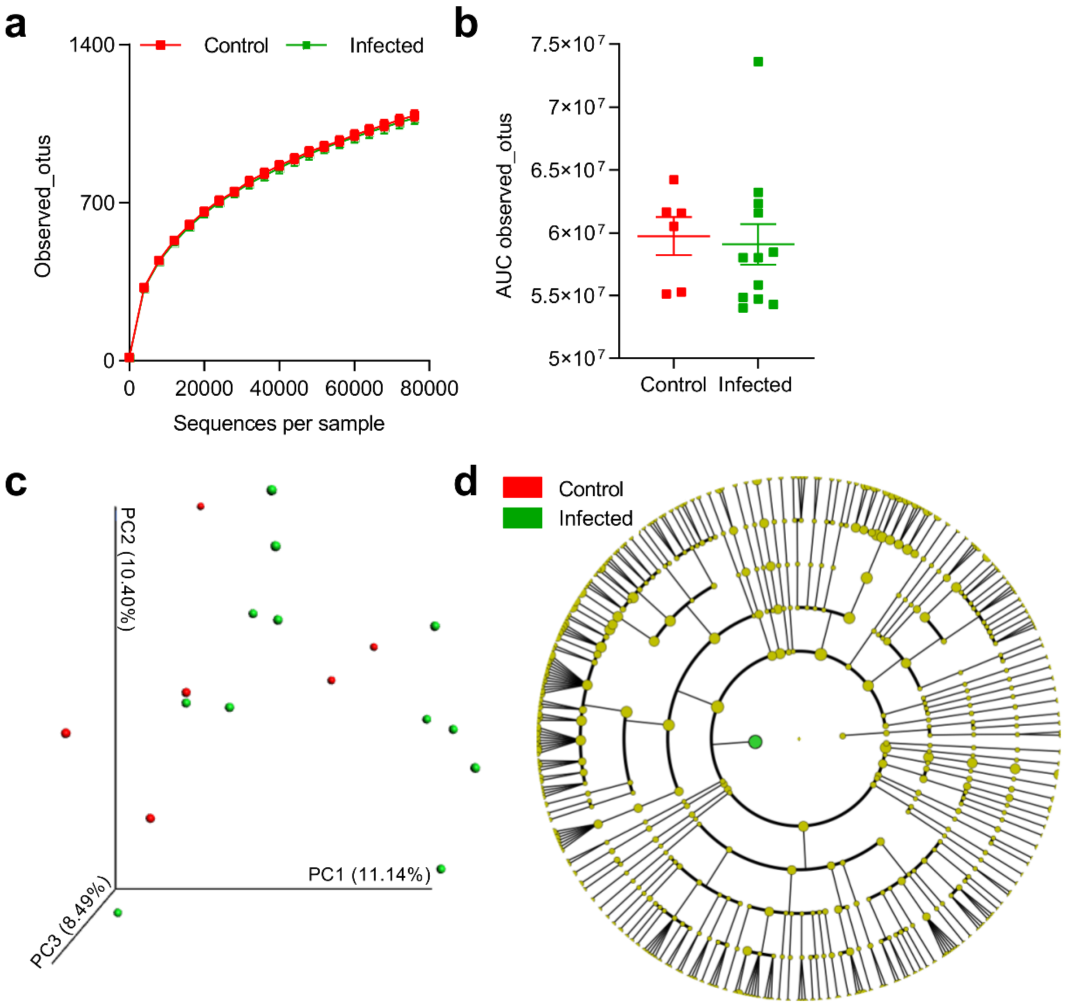

Supplementary Figure 5

Fecal microbiota composition analysis at D0. (a) Alpha diversity was determined by observed OTU measurements according to sequences per sample in feces of control (n=6) and infected (n=12) rats at D0 (i.e. before infection). Statistical analysis was performed with the two-way ANOVA test followed by a Sidak post-hoc test. (b) The area under the curve (AUC) of the observed OTUs for control and infected rats. Statistical analysis was performed with the Mann-Whitney test. (c) Principal coordinates analysis (PCoA) of the unweighted UniFrac distance of control (n=6, red plots) and infected (n=12, blue plots) rats at D0. Significance (p value= 0.109) and the strength of explained variation (R^2= 0.06854) were assessed with Adonis. (d) LEfSE (LDA Effect Size) was used to investigate bacterial taxa that drive differences between control and infected rats. Red, taxa higher in controls; Green, taxa higher in infected rats. No differences were observed.
